# Cross-sectional associations between dietary intake of polyunsaturated fatty acids, physical function, and sarcopenia in community-dwelling older adults

**DOI:** 10.1016/j.jnha.2024.100423

**Published:** 2024-11-29

**Authors:** Hélio José Coelho-Júnior, Alejandro Álvarez-Bustos, Anna Picca, Riccardo Calvani, Leocadio Rodriguez-Mañas, Francesco Landi, Emanuele Marzetti

**Affiliations:** a Fondazione Policlinico Universitario "Agostino Gemelli" IRCCS, Rome, Italy; b Department of Geriatrics, Orthopedics and Rheumatology, Università Cattolica del Sacro Cuore, Rome, Italy; c Biomedical Research Center Network for Frailty and Healthy Ageing (CIBERFES), Institute of Health Carlos III, Madrid, Spain; d Department of Geriatrics, Hospital Universitario de Getafe, Madrid, Spain; e Instituto de Investigación IdiPaz, Madrid, Spain; f Department of Medicine and Surgery, LUM University, Casamassima, Italy

**Keywords:** Nutrition, Malnutrition, Frailty, Muscle atrophy, Muscle strength, Muscle power

## Abstract

**Objectives:**

The present study examined the associations between the dietary intake of polyunsaturated fatty acids (PUFAs), physical function, and the prevalence of sarcopenia in Italian community-dwelling older adults.

**Design:**

Cross-sectional study.

**Setting:**

Unconventional settings across Italy (e.g., exhibitions, health promotion campaigns).

**Participants:**

Older adults (65+ years) who provided a written informed consent.

**Methods:**

Physical function was evaluated according to isometric handgrip strength (IHG) and 5-time sit-to-stand (5STS) performances. Muscle power parameters were estimated based on 5STS values. Sarcopenia was operationalized according to the presence of low physical function (IHG or 5STS) plus low appendicular skeletal muscle mass (ASM), estimated according to calf circumference. A 12-item food questionary was used to estimate the dietary intake of PUFAs, which included omega-3 (α-linolenic acid, eicosapentaenoic acid, docosahexaenoic acid) and omega-6 fatty acids.

**Results:**

Multiple linear regression results indicate negative and significant associations between the dietary intake of α-linolenic acid and muscle power, and between docosahexaenoic acid consumption and ASM. No significant associations were found between PUFAs-related variables and sarcopenia.

**Conclusions:**

Results of the present study indicate that PUFAs-related variables were negatively and significantly associated with physical function and body composition in older adults. Nevertheless, no significant associations were found with sarcopenia. These findings suggest that a more detailed analysis of covariates should be conducted in future investigations that aim to examine the associations between the dietary intake of PUFAs and sarcopenia-related parameters.

## 1 Introduction

Sarcopenia is a neuromuscular disease that is highly prevalent among older adults, with estimates indicating that it affects more than 20% of individuals aged 65+ years [1]. Sarcopenia increases the risk of a wide range of adverse health outcomes, including falls, hospitalization, nursing home placement, and death [[2], [3], [4], [5]]. Additionally, the progression of sarcopenia often coincides with the development of other health conditions, such as frailty, diabetes, and cardiovascular disease [[2], [3], [4], [5]].

According to recent guidelines, sarcopenia should be diagnosed according to the presence of dynapenia and low muscle mass, while eventual deficits in physical performance should be used to estimate its severity [6]. However, muscle power, the capacity to generate muscle strength rapidly [7], declines earlier and to a greater extent than muscle strength [8]. Furthermore, investigations have found that muscle power might be a better predictor of physical independence, functional performance, mobility, and death than muscle strength [[9], [10], [11], [12]]. As such, integrating information regarding muscle power measurements and sarcopenia status provides crucial insights into the physical capacity of older adults.

The genesis of sarcopenia arises from a complex interplay of multiple factors [13,14]. Among these, inadequate nutrient intake has been highlighted as a key contributor in the onset and progression of sarcopenia, with most studies focusing on the role of malnutrition, protein intake, and micronutrients consumption (e.g., vitamin D) [[15], [16], [17]]. Recently, however, there has been growing interest in the impact of polyunsaturated fatty acids (PUFAs) on the development and progression of sarcopenia.

PUFAs are essential fatty acids exclusively obtained from diet sources [18,19]. Omega-3 is one of the main classes of PUFAs and refers to three main types of fatty acids: α-linolenic acid (ALA), eicosapentaenoic acid (EPA) and docosahexaenoic acid (DHA) [18,19]. Omega-6 is another class of PUFAs that are synthetized from linoleic acid, which have the arachidonic acid as its major compound [20].

These molecules are known for their role in cell signaling and structure. Special attention has been given to omega-3 due to its antioxidant and anti-inflammatory effects, at least partly mediated by enhancements in mitochondrial function, likely reducing muscle wasting and neuromuscular losses [20,21]. Furthermore, omega-3 consumption, both as stand-alone and synergistically with amino acids ingestion, might counteract age-related detriments in the neuromuscular apparatus by increasing muscle protein synthesis through the activation of the mechanistic target of rapamycin complex 1 (mTORC1) signaling pathway [20]. On the other hand, omega-6 has pro-inflammatory properties [20], which are generally associated with cell homeostasis, but can also contribute to chronic inflammation and associated diseases, when overconsumed in relation to omega-3 [22,23]. As such, an adequate consumption of omega-3 and omega-6 has been mentioned as a potential strategy to counteract sarcopenia [[18], [19], [20]].

However, despite the premises in favor of omega-3, empirical evidence has provided mixed results [[24], [25], [26], [27]]. Moreover, although results of a recent meta-analysis suggested that the dietary intake of omega-3 might be associated with reduced prevalence of sarcopenia [28], findings were based on a small number of investigations (i.e., 5 studies) and involved substantial heterogeneity. Finally, some criticism has been raised regarding the available evidence, given that many studies did not adjust the results for important confounders and/or did not examine the data using adequate statistical approaches [18].

To expand the knowledge on this theme, the present study was conducted to examine associations between the dietary intake of PUFAs, physical function, including muscle power, and sarcopenia in a large sample of Italian community-dwelling older adults.

## 2 Material and methods

Data of the present investigation were gathered from the Longevity Check-up 8+ (Lookup 8+) project database. Sampling characteristics, procedures, and other results have been published elsewhere [[29], [30], [31], [32], [33]]. Lookup 8+ is an ongoing initiative developed by the Department of Geriatrics of the Fondazione Policlinico “Agostino Gemelli” IRCCS at the Università Cattolica del Sacro Cuore (Rome, Italy). The project was designed to foster healthy and active aging by raising awareness among the general public on the importance of modifiable risk factors for chronic diseases [[29], [30], [31], [32], [33]].

Recruitment was conducted among people visiting public spaces (e.g., exhibitions, shopping centers) and those adhering to prevention campaigns promoted by our institution. As previously described, recruitment activities were carried out in small (<100,000 inhabitants), medium (100,000–250,000 inhabitants), and large cities (>250,000 inhabitants) to achieve a comprehensive geographic coverage of mainland Italy and major islands. In large cities, participants were recruited in different locations to maximize the representation of the sociodemographic characteristics of inhabitants. The Lookup 8+ protocol was approved by the Ethics Committee of the Università Cattolica del Sacro Cuore (protocol #: A.1220/CE/2011) and each participant provided written informed consent prior to enrolment. The manuscript was prepared in compliance with the STrengthening the Reporting of Observational studies in Epidemiology (STROBE) guidelines for observational studies [34].

### 2.1 Participants

From 1 June 2015 to 31 December 2023, 17,576 community-dwelling adults aged 18+ years participated in the study. Exclusion criteria were inability or unwillingness to provide written informed consent, self-reported pregnancy, and inability to perform the physical function tests as per the study protocol. For the present investigation, only people aged 65+ years, with body mass index (BMI) values ≥18.5 kg/m^2^ and no missing data for the study variables, were included, totaling 4461 participants (13,115 excluded).

### 2.2 Data collection

Each participant received a structured interview to collect information on lifestyle habits, followed by measurement of anthropometric parameters, including height and weight. The BMI was calculated as the ratio between body weight (kg) and the square of height (m^2^).

Sarcopenia was identified according to the simultaneous presence of dynapenia and low appendicular skeletal muscle mass (ASM) [6]. Muscle strength was assessed by isometric handgrip strength (IHG) and 5-time sit-to-stand (5STS) testing. Participants underwent IHG testing while sitting comfortably on a chair with their shoulders neutral. The arm being assessed had the elbow flexed at 90° near the torso, and the hand neutral with thumbs up. A maximal contraction was performed over four seconds using a handheld hydraulic dynamometer (North Coast Medical, Inc., Morgan Hill, CA, USA). For the 5STS test, participants had to get up from a chair as quickly as they could while keeping their arms crossed at their chest. Timing started when participants raised their buttocks off the chair and was halted when they were seated at the conclusion of the fifth sit.

Then, 5STS performance was used to calculate absolute muscle power (AMP), relative muscle power (adjusted by body mass, RMP), allometric muscle power (adjusted by height, ALMP), and specific muscle power (adjusted by ASM, SMP) according to the equations proposed by Alcazar et al. [35]:

(1) Absolute muscle power (W) = (Body weight (kg) × 0.9 × g × [height (m) × 0.5 − chair height (m)])/([(5STS test time (s))/(no. of STS repetitions)] × 0.5);
(2) Relative muscle power (W/kg) = (Absolute muscle power (W))/(Body weight (kg));
(3) Allometric muscle power (W/m^2^) = (Absolute muscle power (W))/(Height (m^2^));
(4) Specific muscle power (SMP) (W/kg) = (Absolute muscle power (W))/(ASM).
  ASM was estimated from the calf circumference of the dominant leg. The measure was taken at the largest girth between ankle and knee joints using an anthropometric tape while the participant was in a seated position. ASM was subsequently calculated according to the following formula [36]:
(5) ASM = −10.427 + (calf circumference (cm) × 0.768) − (age × 0.029) + (sex (male = 1, female = 0)) × 7.523.

Sex-specific cutoff points recommended by the European Working Group on. Sarcopenia in Older People 2 (EWGSOP2) were used to categorize muscle strength and ASM values [6].

A food frequency questionnaire (FFQ) was used to collect information on how often in a week participants consumed a standardized portion size of a list of 12 foods, including meat, meat derivatives, fish, eggs, milk, cheese, yogurt, pasta, bread, rice, vegetables, and cereals. Portion size was estimated based on the Italian standard portion reference [37]. Mean daily caloric, protein, omega-6, ALA, EPA, and DHA intakes were calculated by multiplying the consumption frequency of a food item by the protein content of its standard portion [34], then dividing by seven (the days of a week), followed by the sum of all applicable items. Omega-3 consumption was estimated according to the sum of ALA, EPA, and DHA.

Smoking status was defined as follows: current smoker (has smoked 100+ cigarettes in lifetime and currently smokes cigarettes), and no current smoker. Regular participation in physical exercise was considered as involvement in any type of exercise training (e.g., aerobic, resistance). Accordingly, participants were considered either physically active or inactive.

### 2.3 Statistical analysis

The normal distribution of variables was ascertained via the Shapiro–Wilk test. Continuous variables are expressed as the mean ± standard deviation (SD) or percentages. Unadjusted and adjusted linear regressions were conducted to examine associations between PUFAs-related variables and IHG, 5STS, AMP, RMP, ALMP, SMP, and ASM, while unadjusted and adjusted binary logistic regressions were performed to investigate the association between PUFAs-related variables and the prevalence of sarcopenia. The final model was adjusted for age, sex, BMI, energy intake, protein consumption, physical exercise, and active smoking. Significance was set at 5% (*p* < 0.05) for all tests. All analyses were performed using the SPSS software (version 23.0, SPSS Inc., Chicago, IL, USA).

## 3 Results

Data of 4461 older adults (women = 2597, 56%) were analyzed. Main characteristics of study participants are shown in Table 1. Participants had a mean (SD) age of 73.0 ± 6.0 years and an average BMI of 26.1 ± 4.0 kg/m², suggesting that most of them were within normal weight or had overweight. The prevalence of sarcopenia, identified according to the guidelines of the EWGSOP2, was 20.1%. This prevalence is higher than that estimated for the global population by pooled analyzes [1].

**Table 1:** Main characteristics of study participants (*n* = 4461).

Variable	Mean (SD)
Age, years	72.9 ± 5.9
Women, %	56.0
Weight, kg	70.6 ± 13.0
Height, m	1.63 ± 0.11
BMI, kg/m²	26.1 ± 4.0
IHG, kg	27.2 ± 9.7
5STS, s	9.3 ± 0.3
ASM, kg	17.7 ± 5.0
AMP, W	270.7 ± 108.8
RMP, W/kg	3.8 ± 1.1
ALMP, W/m²	98.0 ± 33.0
SMP, W/kg	15.5 ± 5.0
Sarcopenia, %	20.1
Calories intake, kcal	2524.6 ± 759.9
Carbohydrate, g	218.4 ± 71.1
Lipids, g	153.1 ± 67.2
Protein, g	117.6 ± 58.0
SFA, %	31.7
MUFA, %	26.7
Omega-6, %	29.8
Omega-3, %	51.0
ALA, %	8.9
EPA, %	16.0
DHA, %	26.1
Smoking, %	14.0
Physical exercise,%	51.0

5STS = 5-time sit-to-stand; ALA = α-linolenic acid; ALMP = Allometric muscle power; AMP = Absolute muscle power; ASM = Appendicular skeletal muscle mass; BMI = Body mass index; DHA = Docosahexaenoic acid; EPA = Eicosapentaenoic acid; IHG = Isometric handgrip strength; MUFA = Monounsaturated fatty acids; RMP = Relative muscle power; SFA = Saturated fatty acids; SMP = Specific muscle power.

Results of linear and multiple regressions are shown in Table 2. In the unadjusted analysis, ASM and muscle power measures were significantly associated with dietary intake of omega-6 PUFAs, ALA, and DHA. The consumption of ALA was also significantly associated with IHG. EPA and EPA + DHA were significantly associated with 5STS. Furthermore, significant associations between EPA + DHA and ASM were observed.

**Table 2:** Unadjusted and adjusted associations between PUFAs-related variables, physical function, and ASM.

Variables	Unadjusted β	95% CI	P-value	Adjusted β	95% CI	P-value
IHG						
Omega-6	0.01	−0.01 - 0.02	0.461	−0.01	−0.02 - 0.01	0.814
Omega-3	0.01	−0.01 - 0.01	0.760	−0.01	−0.01 - 0.01	0.759
ALA	**0.33**	**0.25-0.42**	**0.001**	−0.07	−0.07 - 0.05	0.837
EPA	−0.09	−0.04 - 0.02	0.601	−0.01	−0.01 - 0.02	0.845
DHA	−0.01	−0.02 - 0.01	0.359	−0.01	−0.01 - 0.01	0.741
EPA + DHA	−0.01	−0.01 - 0.01	0.434	−0.01	0.01 - 0.01	0.775

5STS						
Omega-6	0.01	−0.01 - 0.01	0.480	0.01	−0.01 - 0.01	0.756
Omega-3	−0.01	**−0.01**-**−0.01**	**0.040**	−0.01	−0.01 - 0.01	0.795
ALA	0.01	−0.01 - 0.04	0.240	0.02	−0.06 - 0.05	0.114
EPA	**−0.01**	**−0.02-−0.01**	**0.040**	−0.01	−0.01 - 0.01	0.751
DHA	−0.01	−0.01 - −0.01	**0.020**	−0.01	−0.01 - 0.01	0.573
EPA + DHA	−0.01	**−0.01-−0.01**	**0.020**	0.01	−0.01 - 0.01	0.631

ASM						
Omega-6	**0.01**	**0.01**- **0.02**	**0.008**	−0.01	−0.01 - 0.01	0.555
Omega-3	−0.01	−0.10 - 0.15	0.699	−0.01	−0.01 - 0.01	0.600
ALA	**0.25**	**0.21-0.30**	**0.001**	0.01	−0.01 - 0.02	0.612
EPA	−0.01	−0.03 - 0.01	0.068	−0.01	−0.01 - 0.01	0.080
DHA	−0.01	**−0.02-−0.01**	**0.010**	**−0.01**	**−0.01**-**−0.01**	**0.040**
EPA + DHA	−0.01	**−0.01** -**−0.01**	**0.020**	−0.01	−0.01 - 0.01	0.050

AMP						
Omega-6	**0.11**	**0.02** - **0.47**	**0.029**	−0.01	−0.01 - 0.01	0.895
Omega-3	0.02	−0.01 0.01	0.699	−0.06	−0.17 - 0.04	0.253
ALA	**2.80**	**1.84-3.75**	**0.001**	−0.75	−1.57 - 0.07	0.075
EPA	−0.04	−0.42 - 0.33	0.819	−0.17	−0.50 - 0.14	0.284
DHA	−0.05	−0.25 - 0.15	0.627	−0.07	−0.24 - 0.09	0.402
EPA + DHA	−0.02	−0.01 - 0.01	0.305	−0.05	−0.16 - 0.06	0.357

RMP						
Omega-6	0.01	−0.01 - 0.01	0.585	0.01	−0.01 - 0.01	0.932
Omega-3	0.01	−0.02 - 0.05	0.507	0.01	−0.01 - 0.01	0.569
ALA	**0.01**	**0.01-0.02**	**0.030**	−0.01	−0.01 - 0.03	0.158
EPA	0.01	−0.01 - 0.01	0.347	−0.01	−0.01 - 0.01	0.596
DHA	0.01	−0.01 - 0.01	0.286	0.01	−0.01 - 0.01	0.757
EPA + DHA	0.01	−0.01 - 0.01	0.305	0.01	−0.01 - 0.01	0.699

ALMP						
Omega-6	**0.08**	**0.01- 0.15**	**0.017**	0.01	−0.01 - 0.01	0.826
Omega-3	0.01	−0.02 - 0.05	0.507	−0.01	−0.04 - 0.02	0.655
ALA	**0.67**	**0.38** - **0.97**	**0.001**	−0.17	−0.44 - 0.09	0.202
EPA	0.01	−0.10 - 0.12	0.838	−0.01	−0.12 - 0.08	0.690
DHA	0.01	−0.06 - 0.06	0.977	−0.01	−0.06 - 0.04	0.828
EPA + DHA	0.01	−0.04 - 0.04	0.958	0.01	−0.04 - 0.03	0.779

SMP						
Omega-6	−0.01	−0.01 - 0.01	0.759	−0.01	−0.01 - 0.01	0.865
Omega-3	0.01	−0.01 - 0.01	0.183	0.01	−0.04 - 0.02	0.788
ALA	**−0.08**	**−0.13** -**−0.04**	**0.001**	**−0.05**	**−0.10**-**−0.01**	**0.020**
EPA	0.01	−0.01 - 0.01	0.106	0.01	−0.01 - 0.02	0.762
DHA	**0.01**	**0.01** -**0.01**	**0.040**	0.04	−0.01 - 0.01	0.466
EPA + DHA	0.01	−0.01 - 0.01	0.056	0.02	−0.01 - 0.01	0.559

Bold denotes statistical significance.

5STS = 5-time sit-to-stand; ALA = α-linolenic acid; ALMP = Allometric muscle power; AMP = Absolute muscle power; ASM = Appendicular skeletal muscle mass; BMI = Body mass index; DHA = Docosahexaenoic acid; EPA = Eicosapentaenoic acid; IHG = Isometric handgrip strength; MUFA = Monounsaturated fatty acids; RMP = Relative muscle power; SFA = Saturated fatty acids; SMP = Specific muscle power.

After adjusting the analysis for covariates, negative and significant associations were found between the dietary intake of ALA and SMP, and between DHA consumption and ASM.

Results of unadjusted and adjusted binary regressions are shown in Table 3. In the unadjusted analysis, DHA and EPA + DHA were significantly associated with the prevalence of sarcopenia. However, significance was lost after adjusting the analysis for covariates.

**Table 3:** Unadjusted and adjusted associations between PUFAs-related variables and sarcopenia.

Variables	Unadjusted OR	95% CI	P-value	Adjusted OR	95% CI	P-value
Omega-6	1.002	0.997 - 1.007	0.422	1.000	0.992 - 1.007	0.949
Omega-3	0.295	0.994 - 1.000	0.076	0.999	0.995 - 1.002	0.533
ALA	1.015	0.993 - 1.037	0.173	1.007	0.981 - 1.034	0.604
EPA	0.991	0.983 - 1.000	0.050	0.996	0.986 - 1.007	0.502
DHA	**0.995**	**0.990-1.000**	**0.030**	0.980	0.992 - 1.004	0.471
EPA + DHA	**0.294**	**0.994**-**1.000**	**0.040**	0.999	0.995 - 1.002	0.480

Bold denotes statistical significance.

ALA = α-linolenic acid; CI = Confidence interval; DHA = Docosahexaenoic acid; EPA = Eicosapentaenoic acid; OR = Odds ratio.

## 4 Discussion

The main findings of the present study indicate that the dietary intake of PUFAs-related nutrients was negatively and significantly associated with physical function and body composition in older adults. Specifically, we found that a high consumption of ALA and DHA was inversely associated with SMP and ASM values, respectively. Nevertheless, no associations were found with sarcopenia.

Investigations examining the associations between dietary intake of PUFAs and sarcopenia-related parameters found mixed results. Kim and Park [27], and Bae et al. [26] examined data of the Korea National Health and Nutrition Examination Survey (KNHANES) and found significant associations between EPA and DHA consumption and IHG and muscle mass in women, but not in men. In contrast, no significant associations were observed with ALA in etiher sex [27]. These findings were expanded by Dupont et al. [25], who did not find significant associations between omega-3 PUFAs, EPA, DHA, and EPA + DHA, and 5STS, IHG, or muscle mass in community-dwelling older adults. To complicate things further, Isanejad et al. [24] noted that a high dietary intake of omega-3 PUFAs was associated with better balance and walking speed performances, as well as better results on the Short Physical Performance Battery (SPPB).

These contradictory results, including those found in the present study, might be attributed to differences in sample characteristics and sizes, simultaneous intake of macronutrients, dietary assessment tool, lack of adjustment for important confounders, and the statistical approach employed for data analysis [18]. Notably, a high dietary intake of omega-3 PUFAs might be accompanied by an increased consumption of other types of fats (e.g., saturated fats), which has been associated with the presence of sarcopenia [38,39].

Indeed, some classical Italian recipes rich in sources of omega-3 PUFAs by including fish (e.g., anchovies) and seafood (e.g., oysters) might also involve a high content of total and saturated fats due to preparation methods (e.g., fry), accompaniments (e.g., cheese), and/or food characteristics (e.g., content of macronutrients), such as *il fritto misto di pesce* (mixed fried fish)*, risotto al salmone* (salmon risotto), and pizza. However, a complementary analysis indicated no significant associations of the dietary intake of total fats and SFA with SMP or ASM (Supplementary material [SM] 1).

An alternative explanation is based on the nature of the covariates used to adjust data analysis. For instance, the practice of physical exercise was included in the final model, given its association with healthy lifestyle habits [40] and sarcopenia [41]. However, specific exercise regimes (e.g., high intensity resistance training, power training) might be required to maintain or improve SMP and ASM in older adults [42]. Therefore, future analyses providing a more detailed examination of participants’ physical exercise characteristics are warranted.

Another element that deserves attention is that the Italian cooking methods and patterns of dietary intake of fish and seafood are regionally determined, with southern regions exhibiting higher consumption levels compared to the rest of the country [43,44]. This suggests that differing results could emerge if regional specificities were considered in the analysis. Nevertheless, since nutritional habits were assessed through an FFQ and participants’ regions were not recorded, the conduction of more detailed analyses was not feasible.

Findings of the present study indicate that the dietary intake of PUFAs was not significantly associated with the prevalence of sarcopenia in Italian community-dwelling older adults. These findings are in line with the observations of Das et al. [45] in Australian older adults. On the other hand, Okamura et al. [46] and Yang et al. [47] found that the intake of omega-3 PUFAs was inversely associated with sarcopenia prevalence. These findings might suggest that the dietary intakes of PUFAs of our examined population were not sufficient to promote changes at cellular level. In fact, endogenous sources are not sufficient to promote the synthesis of omega-3 molecules, requiring an adequate exogenous intake of PUFAs [19].

Other important limitations, in addition to those aforementioned, should be mentioned for a better interpretation of our results. First, our sample involved relatively young community-dwelling Caucasian older adults with apparently preserved physical function and adequate caloric intake. The fact that higher consumption of nutrients does not appear to be associated with improved health outcomes in healthy individuals [48] suggests that extrapolating our results to individuals in different conditions should be done with caution. Second, participants were evaluated while they were attending an event. Thus, the possibility that the evaluation setting could have influenced physical performance results cannot be ruled out. Third, information on chronic diseases (e.g., osteoarthritis) or medications (e.g., corticosteroids) that could impact musculoskeletal health was not available. The collection of a detailed medical history would substantially increase the duration of the assessments, making them unsuitable for the unconventional settings where the research is conducted. Fourth, the prevalence of participants on PUFA supplementation was not recorded. This information is important given that PUFA supplementation is commonly recommended in older adults. Fifth, lower limb muscle power and ASM were estimated through equations rather than being measured directly. Although muscle power equations have been validated, the use of a linear encoder could provide better estimations of muscle power [49]. Sixth, the use of FFQs carries important limitations, such as recall bias and closed-ended questions, which are especially relevant when applied to older populations [50]. Furthermore, the FFQ used in the present study included the weekly consumption of only 12 food groups, did not explore the consumption of snacks, beverages, and alcohol, and was not validated in large samples sizes against standard methods (e.g., diet diary). As such, future studies employing other dietary assessment methods should be conducted to confirm our findings. Finally, the cross-sectional design of this study does not allow any inference to be drawn on the time course of changes in the variables considered and on cause–effect relationships.

## 5 Conclusions

Results of the present study indicate that PUFAs-related variables were negatively and significantly associated with physical function and body composition in older adults. Nevertheless, no associations were found with sarcopenia. These findings suggest that a more detailed analysis of covariates should be conducted in future investigations that aim to examine the associations between the dietary intake of PUFAs and sarcopenia-related parameters.

## CRediT authorship contribution statement

Conceptualization: HJCJ, AAB, LRM, EM; Methodology: HJCJ, AAB, AP, RC, LRM,FL, EM; Formal analysis: HJCJ, AAB, AP, EC, EM; Investigation: HJCJ, AAB, EM, AP, RC; Data curation: HJCJ, AAB, EM, LRM, FL; Writing-original draft preparation: HJCJ, AAB, EM, AP, RC; Writing-review and editing: HJCJ, LRM, FL, EM; All authors have read and agreed to the published version of the manuscript.

## Funding

This research did not receive external funding.

## Declaration of Competing Interest

The authors declare that they have no known competing financial interests or personal relationships that could have appeared to influence the work reported in this paper.

## Acknowledgments

The article processing charge was funded by the Italian Ministry of Health (Ricerca Corrente 2024).

## Data availability statement

Raw data files are available from Prof. Landi upon reasonable request.

## Declaration of generative AI in scientific writing

No Generative AI or AI-assisted technologies were used in the writing process.
