# Supplementary material for: Cross-sectional associations between dietary intake of polyunsaturated fatty acids, physical function, and sarcopenia in community-dwelling older adults
Source: J Nutr Health Aging. 2024 Nov 29;29(1):100423. doi: 10.1016/j.jnha.2024.100423 (PMC12179987; doi:10.1016/j.jnha.2024.100423)
Supplement: Supplementary file 1 [file mmc1.doc]

| Supplementary Material 1. Multiple linear regression for the association between fatty acids consumption and sarcopenia parameters | | | | | |
| --- | --- | --- | --- | --- | --- |
| Variables |  | Adjusted β | 95% CI | P-value |  |
| SMP |  |  |  |  |  |
| Total fatty acids | | 0.01 | -0.01, 0,01 | 0.589 |  |
| SFA |  | 0.01 | -0.01, 0.01 | 0.462 |  |
|  |  |  |  |  |  |
| ASM |  |  |  |  |  |
| Total fatty acids | | **0.139** | **0.01, 0.01** | **0.011** |  |
| SFA |  | -0.011 | -0.01, 0.01 | 0.095 |  |
| ASM= Appendicular skeletal muscle; CI= Confidence interval; SMP= Specific muscle power; SFA= Saturated fatty acids; | | | | |  |
